# Low Earth Orbit Communication Satellites: A Positively Disruptive Technology That Could Change the Delivery of Health Care in Rural and Northern Canada

**DOI:** 10.2196/46113

**Published:** 2025-04-30

**Authors:** Douglas Hamilton, Sandeep (Sonny) S Kohli, Paul McBeth, Randy Moore, Keltie Hamilton, Andrew W Kirkpatrick

**Affiliations:** 1 General Internal Medicine Faculty of Medicine University of Calgary Calgary, AB Canada; 2 Department of Electrical Engineering Faculty of Engineering University of Calgary Calgary, AB Canada; 3 Space Advisory Board Minister of Innovation, Science and Economic Development Government of Canada Ottawa, ON Canada; 4 Intensive Care and Internal Medicine Oakville Trafalgar Hospital McMaster University Oakville, ON Canada; 5 Department of Critical Care Medicine Faculty of Medicine University of Calgary Calgary, AB Canada; 6 Department of Surgery Faculty of Medicine University of Calgary Calgary, AB Canada; 7 Department of Mechanical and Manufacturing Engineering Faculty of Engineering University of Calgary Calgary, AB Canada; 8 Faculty of Health University of Lethbridge Lethbridge, AB Canada; 9 TeleMentored Ultrasound Supported Medical Interventions Research Group Faculty of Medicine University of Calgary Calgary, AB Canada

**Keywords:** telemedicine, telementored medicine, space medicine, virtual medicine, medical informatics, low earth orbit satellites, rural, Canada, remote, stress, virtual care, medical care, utility, COVID-19, patient, availability, satellite, mobile phone

## Abstract

Canada is a progressive nation that endeavors to provide comprehensive, universal, and portable health care to all its citizens. This is a challenge for a country with a population of 40 million living within a land expanse of 10 million km2 and where 18% live in rural or highly remote locations. The combined population of Yukon, Northwest Territories, and Nunavut is only 128,959 (0.32% of the population), living within 3.92 million km2, and many of these citizens live in isolated communities with unique health needs and social issues. The current solution to providing health care in the most remote locations has been to transport the patient to the health care provider or vice versa, which incurs considerable financial strain on our health care system and personal stress to the patient and provider. The recent global deployment of low Earth orbit communication satellites (LEO-ComSats) will change the practice and availability of online medicine everywhere, especially in northern Canada. The deployment of LEO-ComSats could result in disruptive but positive changes in medical care for underserved communities in remote geographic locations across Canada. LEO-ComSats can be used to demonstrate online medical encounters between a patient and a doctor in Canada, separated by thousands of kilometers. Most certainly, the academic medical centers in lower Canada could perform online telementored medical care to our northern communities like the remote care provided to many Canadians during the COVID-19 pandemic. An online health care model requires effective design, testing, and validation of the policies, standards, requirements, procedures, and protocols. Although the COVID-19 pandemic was the initial prime mover across all of Canada in the use of online medical encounters and creating rapidly devised reimbursement models, it was nonetheless created reactively, using real-time managerial fiat and poorly defined procedures based on minimal pedagogical experience, which made it “difficult to prove it was universally safe.” It is essential to proactively derive the medical policies, standards, and procedures for telementored medicine and “prove it is safe” before LEO-ComSat technology is ubiquitously deployed in northern Canada. This viewpoint was written by subject matter experts who have researched online and internet-based medicine for many years, sometimes 3 decades. In many cases, a literature review was not necessary since they already had the articles in the bibliography or knowledge in their possession. In many cases, internet search engines (ie, Google or PubMed) and Canadian government documents were used to provide corroborating evidence.

## Introduction

If this is a medical emergency, please hang up and dial 911 or go to the nearest emergency room.

How many patients have heard this recorded after-hours message? Unfortunately, in Canada, the emergency department can be 2000 km away, and when the transported patient arrives, very little acute or longitudinal medical data are available.

Canada is a progressive nation that endeavors to provide comprehensive, universal, and portable health care to all its citizens. This is a challenge for a country with a population of 40 million living within 10 million km^2^ and where 18% live in rural or highly remote locations [1]. The combined population of Yukon, Northwest Territories (NWT), and Nunavut is only 128,959 (0.32% of the population), living within 3.92 million km^2^, and many of these citizens live in isolated communities with unique health needs and social issues [2]. Even with the allocation of large per capita expenditures, Canadian health care providers face the almost insuperable task of providing universal and comprehensive remote care.

In many cases, the health care providers in remote communities are medical technicians or nurses treating various chronic diseases and acute injuries with little real-time support. These isolated health care workers provide acute and chronic disease care as well as illness and injury prevention and management for these underserved and vulnerable populations, including older adults and youth, patients with lower socioeconomic status, and those with developmental and functional disabilities within a wide range of racial, ethnic, linguistic, and cultural diversity. Despite the hard work of these medical care workers, these remote patients are at greater risk of poorer health outcomes and have experienced challenges and inequity in access to Canada’s universal health care system [3].

First Nation, Métis, and Inuit people in Canada experience disproportionate rates of traumatic injury and critical illness, reported as 6.5 times greater when compared with the Canadian average (and is even worse in some jurisdictions) [4,5]. Although the determinants of First Nation, Métis, and Inuit communities’ health outcomes are highly complex and multifactorial, remote geography and transportation are significant contributors. Independent of race or ethnicity, patients injured in rural settings have a 50% increased mortality after motor vehicle crashes and worse functional outcomes. Deaths from all injuries for male and female individuals were 4 times higher per capita in the NWT and Nunavut, compared with Ontario [6]. Nearly half a century of research has shown that consolidating expertise at geographic sites known as trauma centers improves outcomes. However, by air transport, 20% of Canadians live over an hour away from a trauma center. Therefore, northern Canada, comprising 41% of our land mass, lacks reliable access to definitive trauma care [7].

In these remote settings, many deaths are from conditions that are both predictably common and potentially treatable if basic therapies and interventions are available. Such therapies include simple direct pressure for hemorrhage control, airway management, percutaneous interventions for a collapsed lung, and full intracavitary surgical interventions. A critical distinction between traumatic injury and many other medical conditions is the critical element of time. The same initial interventions, such as drainage of a hemothorax, intubation, or external hemorrhage control, that may completely manage a situation early may be futile if applied too late to a physiologically compromised, dying patient. Trauma care also must have a practical side, such that in mass casualty or austere situations, psychologically burdensome decisions regarding futility or excessively resource-consumptive situations can be shared between first responders and remote specialists. To provide advanced care, the key ingredients to facilitate such interventions are appropriate equipment and supplies and appropriately trained caregivers. Realistically, it is often far easier logistically to have equipment available than to have providers use it properly, especially if the equipment is as basic as a needle or pack of gauze.

**Figure 1:**
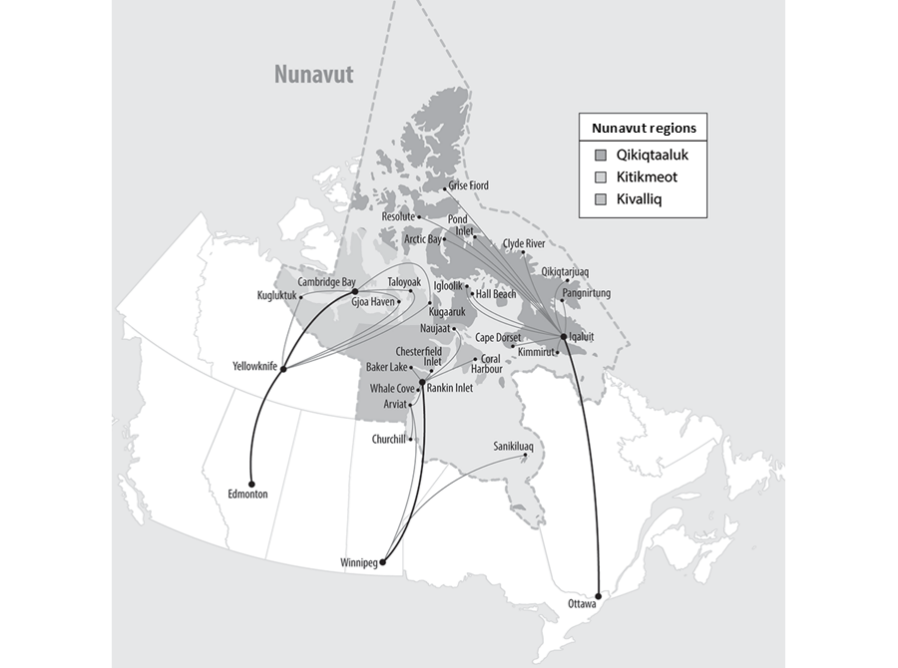
There are air links between Nunavut communities and the locations where residents may be transported (Edmonton, Winnipeg, and Ottawa), if they cannot receive the required health care in their community [8].

For example, delivering health care services in Nunavut is difficult for many reasons, such as the size of the territory, dispersion of the small population, unpredictable weather, and a dependence on air transportation [8]. Nunavut’s 25 communities are isolated and dispersed across Canada’s largest territory or province. Nunavut’s communities can only be accessed year round by air transportation, a major resource for health care delivery (Figure 1) [8].

The current solution to providing health care in these remote locations has been to transport the patient to the health care provider or vice versa, which incurs considerable financial strain on our health care system and personal stress to the patient and provider. One of the most stressful concerns is the danger and futility of unnecessary transport and the higher crash rates of medical evacuation flights [9]. Recent advances in diagnostic imaging and communication technology, combined with the unique medical solutions in the area of telementored medicine (TMED) created by the authors, have brought forth a solution that will help Canada deliver superior health care to our remote populations.

The recent global deployment of low Earth orbit communication satellites (LEO-ComSats) will change the practice and availability of online and internet-based medicine everywhere, especially in northern Canada. The rapid deployment and implementation of LEO-ComSat technology is demonstrated by the use of Starlink terminals to recover the internet capability for Ukrainians during the Russian invasion of their country [10]. It is important to understand that LEO-ComSats will provide low-latency, high-bandwidth, and inexpensive satellite communication to support cell phones and web-based applications for the first time anywhere in the world. These services include Facebook, Netflix, Zoom, Google, YouTube, broadcast television, high-performance internet video games, etc, and almost any website in a manner similar to that provided by any cable service.

This study was written by subject matter experts who have researched internet-based medicine for many years, sometimes 3 decades. In many cases, a literature review was not necessary since they already had the articles in their bibliography or knowledge in their possession. In many cases, internet search engines (ie, Google or PubMed) and Canadian government documents were used to provide corroborating evidence.

### The New Paradigm: Post–COVID-19 Telemedicine

Canada has a long-standing history of using telemedicine, starting with Dr Max House, a founder of Memorial University’s medical school and considered a telemedicine pioneer worldwide. There are many definitions of telemedicine and various synonymous terms, like telehealth, eHealth, connected health, and health telematics. All these definitions and synonyms include at least 2 key components: geographic separation between medical expertise and the patient and technology-mediated interaction. A suggested working definition is that “telemedicine uses information and communications technologies and infrastructure to mediate medical care transactions at a distance.” In total, 3 basic modalities of telemedicine interaction are classified by latency or delay of telecommunications: store-and-forward telemedicine, high-latency telemedicine, and real-time (synchronous) telemedicine (see Textbox 1).

In the past, telemedicine projects and research usually resulted in some peer-reviewed publications and reports, but operational deployment into a health care system was rare. Typically, this was because of the medical-legal issues of delivering health care remotely away from the bedside or poor financial health care provider reimbursement strategies. Recent challenges created by the COVID-19 viral pandemic have caused many care providers to perform telemedicine encounters over the internet or phone rather than in-person consultations.

Given the recent necessity of providing online and internet-based health care delivery because of the COVID-19 pandemic, the ability to perform many medical encounters remotely is becoming possible. As this new viral pandemic spread, payers around the globe rapidly altered fee schedules and medical care provider reimbursement strategies to encourage online visits to reduce the risk of viral transmission, protect high-risk patients with chronic diseases, provide medical attention, and conserve personal protective equipment resources. During Canada’s first wave of the COVID-19 pandemic, provincial health insurance programs quickly created new billing codes that permitted telephone and web-based technology (eg, Skype and Zoom) to perform online medical encounters [11]. Unfortunately, there were many regions in Canada where these online medical encounters were difficult, if not impossible, because of insufficient telecommunication or internet infrastructure. On July 8, 2022, Canada experienced a nationwide loss of a major communication provider for more than 1 day, affecting millions of Canadians in transit, cell phones, cable, GPS maps, banking, ATM, and internet access [12]. Physicians and patients could not communicate, and 911 services were unavailable in many cities. With the shift to delivering virtual medical encounters to ameliorate the effects of COVID-19, the failure of an extensive communication network created chaos in all medical communities.

Bhatia et al [11] reported that the use of online and internet-based care by Ontario residents (population of 14.6 million) increased from 1.6% of all ambulatory visits in the second quarter of 2019 to 70.6% in the second quarter of 2020 due to the COVID-19 pandemic. Physicians who provided 1 or more online and internet-based medical encounters per year increased from 7% in the second quarter of 2019 to 85.9% in the second quarter of 2020 [11]. The proportion of Ontarians who had at least one virtual visit increased from 1.3% in 2019 to 29.2% in 2020 [11]. Older patients were the most significant consumers of online or telephone-based care, most likely because of their increased risk of exposure to COVID-19 outside their residences. This reflects a massive shift in our health care delivery paradigms during the COVID-19 pandemic to online or telephone-based care telemedicine encounters.

This broad acceptance of online care in many nations became essential because in-person encounters were initially impossible due to the closure of clinics and medical facilities from COVID-19 lockdown and quarantine policies to “flatten the curve.” After the intended 15- to 30-day lockdown period, many nations continued to demand various forms of isolation and quarantine for the next 2 years. During this time, online care usage in the United States plummeted from its peak of 69% of all doctor-patient visits in April 2020 [13]. This was observed in many other nations across Europe and Asia and was primarily due to medical health care systems finding a way to open clinics and facilities, control COVID-19 exposures, and provide in-person encounters. In many countries, barriers to implementing virtual care after COVID-19 still exist in regulations, payment regimes, medical and legal protection, and patient acceptance. Due to COVID-19, the online and internet-based care floodgate has now been opened, and any nation seeking to raise health care quality, increase access, and lower costs should be expanding, not contracting, the use of internet and telephone-based care.

The acceptance of online and internet-based care in remote Canada is essential if the requirements of the Canada Health Act are to be met for every Canadian.

**The 3 primary modalities of telemedicine interaction.**

- Store-and-forward telemedicine: Data are collected and stored offline and transmitted (or forwarded) to the destination site later. Familiar store-and-forward interactions include email, text messaging, and voice mail, all used quite effectively in contemporary home and office environments. A store-and-forward consultation may have a latency of more than 24 hours; however, this is not much different from referring a patient to a specialist who may not see the referred patient for days or weeks. Radiology, dermatology, ophthalmology, and pathology are clinical specialties widely adopted by store-and-forward telemedicine.
- High-latency telemedicine: Latency is the delay in a round-trip data transmission, usually measured in milliseconds. Geostationary satellite systems have a median latency of nearly 600 milliseconds, making them an unsuitable replacement for cable or fiber systems. This high latency environment prevents web-based servers from effectively supporting real-time applications. It often renders the internet unusable except for file transfers, bidirectional video and audio streaming, and email. The 600-millisecond delay of a geostationary satellite system interferes with many social media websites and video conferencing systems that use spectral and temporal compression algorithms.
- Real-time (synchronous) telemedicine: This modality involves little or no perceptible latency. Real-time interactions include full-motion videoconferencing or using interactive web-based applications such as an electronic medical record system found in most hospitals. Real-time telementored medicine involves the interaction between a presenter (often a nurse or medical assistant) attending to a patient and the remote medical specialist. This mode of communication has a latency that permits the uninhibited relationship between web-based client and server communication and is usually confined to terrestrial phone and data networks but will soon include low Earth orbit (LEO) communication satellite systems, such as the Telesat-LEO “Lightspeed,” SpaceX “Starlink,” OneWeb, and Amazon. Using a network of several hundred to thousand intercommunicating LEO satellites, the average latency can be reduced to approximately 30 milliseconds. These LEO communication satellite constellations (LEO-ComSats) allow remote medical providers to access electronic medical records (EMR) and radiological picture archiving and communication systems (PACS) as if they were in a large tertiary care hospital. With this level of latency, current telerobotic surgery technologies can be enabled anywhere in the world.

### Remotely Guided Telemedicine Interventions: TMED

#### Overview

Over the past half-century, geosynchronous satellite communication technology has been used to transmit diagnostic imaging for remote interpretation. In many cases, the person acquiring the image is trained and certified to use the imaging equipment. This has been done to ensure that the image quality is clinically useful for diagnosis and treatment.

Over the past 3 decades, ultrasound imaging technology has evolved from expensive, large, refrigerator-sized machines to small, affordable laptop or handheld devices, and its applications have expanded to include almost all areas of medicine. With the ability to transmit near real-time video from a remote location through high-latency satellite or low-latency, land-based communications, remotely guided ultrasound can be used for preventive screening or help diagnose illness or injury using nonmedical personnel.

**Figure 2:**
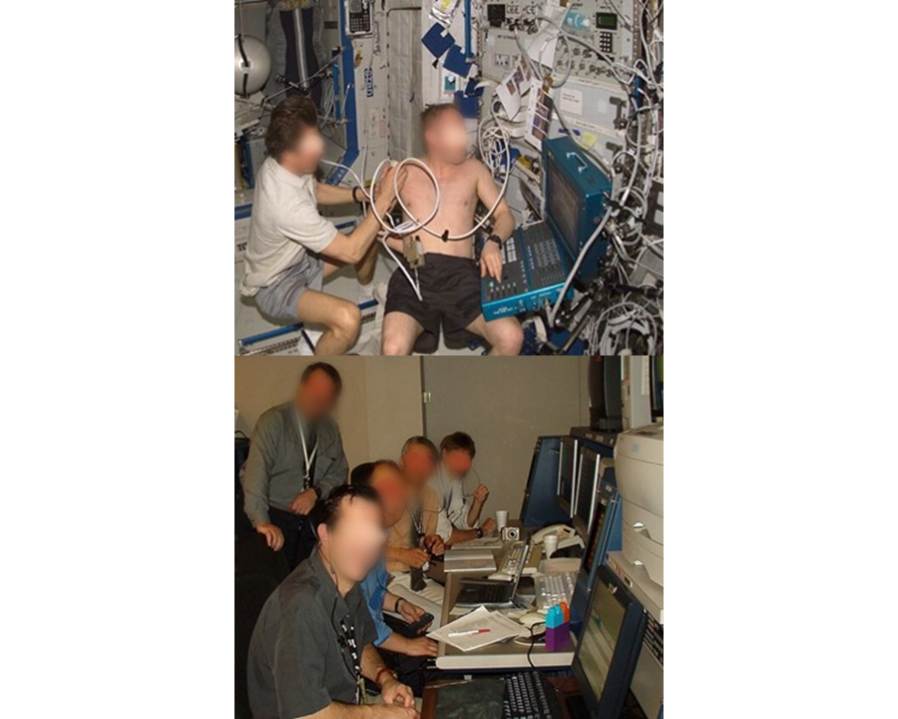
Gennady Padalka performed an ultrasound exam on Mike Fincke’s shoulder (2004) during Expedition 9 on the International Space Station (Upper panel) [17]. Padalka is remotely telementored by the telementored ultrasound (TMUS) team in Mission Control, Houston. (Lower panel).

The problem of having only limited medical resources in a remote location in northern Canada is no more extreme than aboard the International Space Station (ISS). The use of remotely guided ultrasound imaging was pioneered by the TMED team with NASA [14-21], where it is the only imaging modality currently available on the ISS. The concept of remote guidance of untrained ultrasound operator astronauts was initially met with some skepticism; however, recent studies performed on the ISS have demonstrated the ability to acquire diagnostic quality real-time ultrasound images obtained using novice ultrasound operators (Figure 2) [14-16]. To date, almost all anatomical areas of the human body have been studied in space using this technique (more than 500 sessions involving more than 80 astronauts and 30 different specialists).

**Figure 3:**
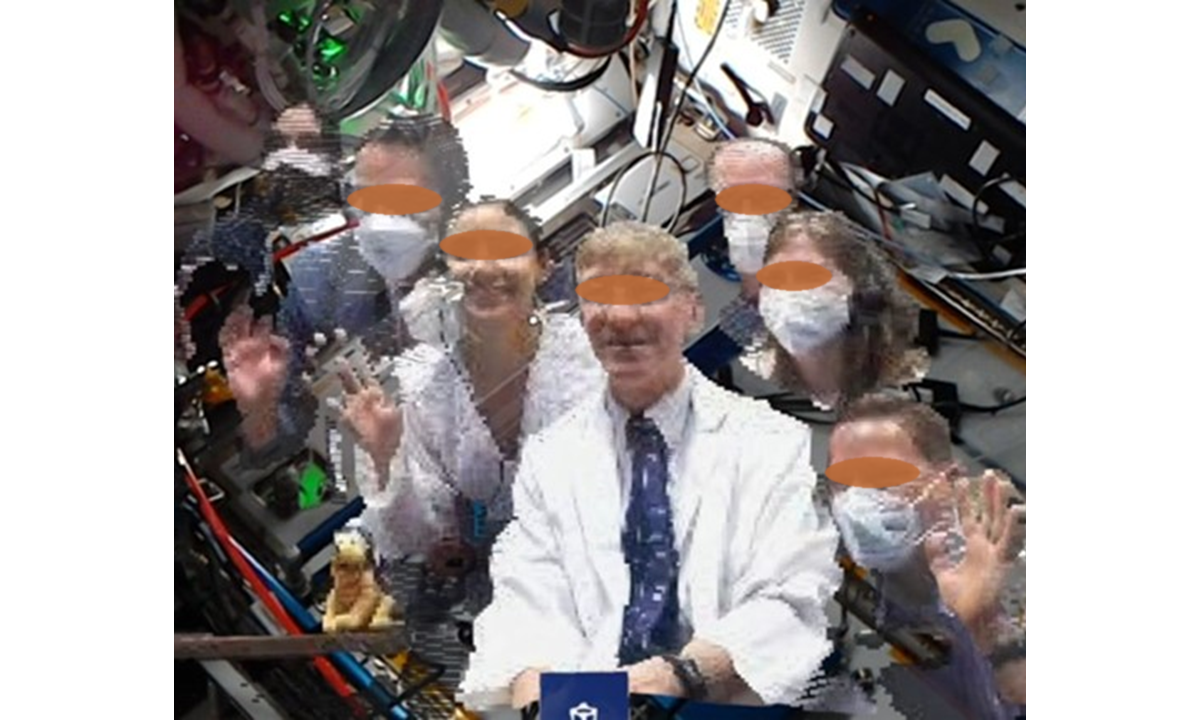
Hologram of medical professionals on the International Space Station (ISS) on October 8, 2021 [19].

This concept was further proven during the Olympics, where nonmedical athletic trainers in Turin, Beijing, London, and Vancouver were guided in real-time through an ultrasound exam by experts at the Henry Ford Hospital in Detroit (more than 300 sessions involving more than 100 athletes) [18]. Athletes from several countries were examined using this concept and returned to win medals after experts in the United States discussed their cases. Other telementored examinations performed on the ISS include ocular ultrasound imaging and fundoscopy. In 2021, a team of hologram doctors was “holoported” to space to visit astronauts on the ISS (Figure 3) [19]. NASA flight surgeon Dr Josef Schmid led the hologram medical teams and the first humans to be “holoported” from Earth to space. This technology can be adapted to facilitate patient encounters in remote locations in northern Canada with physicians in large medical centers anywhere in Canada.

#### The First Canadian Telementored Ultrasound Project (2006)

Canada has extensive fiber-optic “backbones” that extend practically everywhere south of 60° latitude. Above this latitude, connectivity becomes more dependent on commercially provided geostationary satellite communication, which becomes difficult for more northern communities because most geosynchronous satellite systems do not serve them.

**Figure 4:**
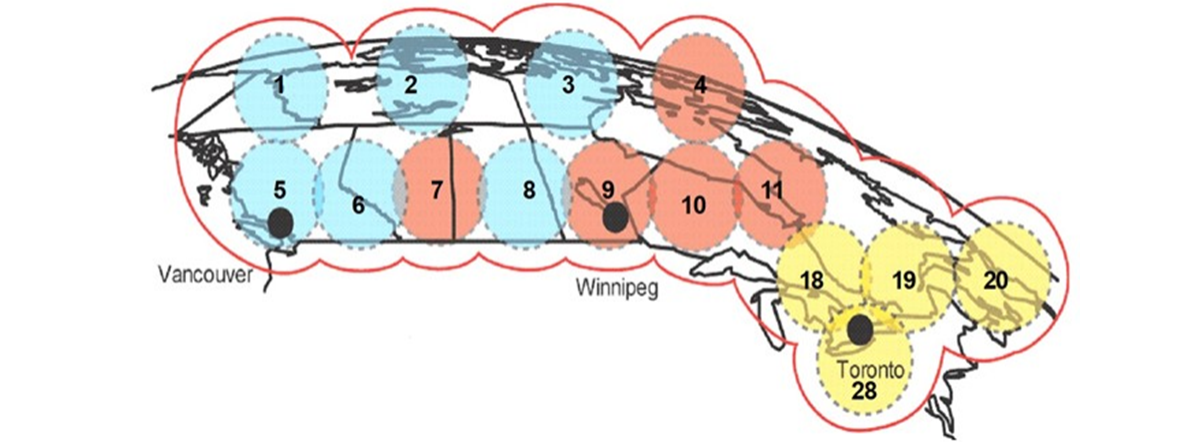
Beams from the Anik F2 Satellite. The 3 beams (designated Beams 1, 2, and 3) are home to the Vancouver gateway, while Beam 4 uses the Winnipeg gateway. These gateways enabled any base station in northern Canada to connect to the data servers such as email and streaming video services. Still, their inherent communication latency prevents end users from assessing real-time, web-based servers and services [20].

In July 2004, the Canadian Space Agency (CSA) launched the 5900-kilogram Anik-F2 satellite, which, at that time, was one of the largest, most powerful communications satellites ever built. With its use of Ka-band technology, low-cost 2-way satellite delivery was made available for telementored medicine, teleteaching, teleworking, streaming video, and e-commerce in many remote regions of Canada. The Government of Canada had initially dedicated the transponders covering the Canadian Arctic for use by researchers and government programs and agencies. However, they were eventually leased to commercial communication companies. The capacity available for the TMED team between 2005 and 2009 was in the 4 arctic Anik F2 Canadian beams (see Figure 4).

The first project performed by the TMED team with Telesat Canada was funded in 2005 by the Space Technologies Development Program [20] of the CSA to test the concept of transmitting real-time, remotely guided ultrasound from Banff to Calgary during emergency trauma resuscitations using terrestrial telephone internet service providers. These ultrasound sessions (n=15) revealed 2 cases of intraperitoneal fluid; in both instances, follow-up computed tomography scanning was recommended, which confirmed significant injuries requiring hospitalization [21,22]. In one remarkable case, the remote ultrasound revealed the source of exsanguination was intraperitoneal, which prompted a direct admission to the operating room, bypassing the emergency department trauma team for immediate surgery. The first remotely guided ultrasound diagnosis of a pneumothorax was also conducted [22].

The next phase of the TMED project used the Anik-F2 to perform remotely guided obstetrical ultrasound imaging from northern communities in Nunavut (Iqaluit: n=12; Rankin Inlet: n=10; and Cambridge Bay n=10) [20]. In real time, these sessions were conducted using a remote sonographer to transmit images to a radiologist in Ottawa. The diagnostic quality of these images enabled the radiologist in Ottawa to perform a routine fetal and maternal wellness ultrasound examination and investigate emergent, possibly life-threatening obstetrical conditions (eg, follow-up of spontaneous abortion or ruling out products of conception).

#### Telementored Mental Health and Addiction Services

Telementored psychiatric examinations have proven to be remarkably effective during the COVID-19 pandemic, and this includes addiction recovery programs and online group support sessions such as Narcotics Anonymous, Alcoholics Anonymous, or Al-anon. Mental health is essential to overall wellness and well-being, and taking care of mental health in remote, isolated northern communities is extremely important. LEO-ComSats could help provide mental health support through online websites and phone apps. The NWT has several programs for mental health, such as Abiliti-Cognitive Behavioral Therapy, Child and Youth TeleLink Program, Community Counselling Program, Employee and Family Assistance Program or Life Speak, Opioid Management Treatment Program, Strongest Families Institute, and Crisis Lines. LEO-ComSats can help ubiquitously support deploying these capabilities everywhere in northern Canada.

Currently, in remote areas of Canada, many seeking treatment services for addiction must be flown into more metropolitan areas in neighboring provinces to seek treatment and return home after a residential program [23]. This has resulted in the NWT having 4 times the hospitalization rate for addiction-related admissions compared with any other province or territory in Canada at 2015 per 100,000 people annually [24].

The NWT government, working with third-party developers, provides anonymous and confidential peer support web-based applications that help support mental health and addiction support, such as Edgeworth Health Network (EHN) Outpatient Services Addictions Virtual Aftercare. EHN Outpatient Services provides an abstinence-based, online, and internet-based maintenance program that can be accessed following addiction treatment.

Currently, these mental health and addiction services are available to those in the NWT and other remote areas of Canada. Still, they rely heavily on access to the internet or cellular service. More importantly, only supporting abstinence when it comes to substance use is not holistic enough, especially in communities that have limited resources for mental health and addiction services.

Using LEO-ComSat telemedicine to provide real-time access to specialists in substance use and mental health could allow for the introduction and sustainability of mental health services, harm reduction practices, and addiction services in very remote communities. A qualitative study conducted at the busiest supervised consumption site in North America (ARCHES, Lethbridge, Alberta) highlighted that offering options for substance use and a reduction in use was what most people seeking these services felt was effective instead of traditional abstinence-based treatment [25]. In addition, First Nations, Métis, and Inuit people make up a large portion of rural and remote populations in these areas and should be included in all planning related to telemedicine projects, ideas, and implementation in their communities as they have the best understanding of their communities wants and needs [26].

The hope of using LEO-ComSats to facilitate real-time telemedicine encounters to bridge this mental health and addiction gap and eventually support not only abstinence-based posttreatment programs but also proactive preventative harm reduction initiatives could be the innovative and realistic way to address high rates of substance use and mental health crises in the most rural and remote areas of Canada [26].

#### Cloud Computing and Artificial Intelligence to Improve Computer-Aided Diagnostics and Performance for Telementored Encounters

With advancements in machine learning and artificial intelligence (AI), integrating computer-aided remote diagnostics and human performance evaluation will be essential in improving diagnostic accuracy and end-user performance and providing a platform for future automation of diagnostics and interventions. LEO-ComSats have the potential to enable reliable access to cloud computing resources, which are becoming increasingly used in the health care system. Cloud computing allows applications to provide computing infrastructure to many users with dynamically changing requirements [27]. Notably, cloud computing allows an individual with internet access to use computing power that is not present on local hardware. This becomes especially useful when deploying powerful, computationally intensive AI algorithms. In 2017, the US Food and Drug Administration approved the first clinical cloud-based deep learning product, Cardio DL (from Arterys) [28]. This system analyzes magnetic resonance images using AI algorithms to provide clinically relevant and quantitative data on heart function [28]. In the years since, the importance of AI in health care has been realized as more evidence indicates AI’s potential for cost savings and its ability to perform on par or better than humans at tasks such as image analysis and the characterization and prognosis of the disease [29]. As a result, access to these cloud computing AI services can supplement remote health care providers with advanced diagnostic tools, potentially providing diagnoses from low-cost devices connected to the internet. Ultimately, LEO-ComSats can link low-cost, remote hardware with vast computational power, bringing advanced health care–related AI to the most remote locations on the planet.

#### Telementored Electronic Medical Records and Patient Support

A significant challenge to providing medical care in northern Canada has been the inability to reliably maintain the same patient’s comprehensive and current medical records over thousands of kilometers. As the LEO-ComSats provide low-latency, high-bandwidth communication to practically every location in Canada, but more importantly, northern Canada, the ability to support ubiquitous internet connectivity is now possible.

**Figure 5:**
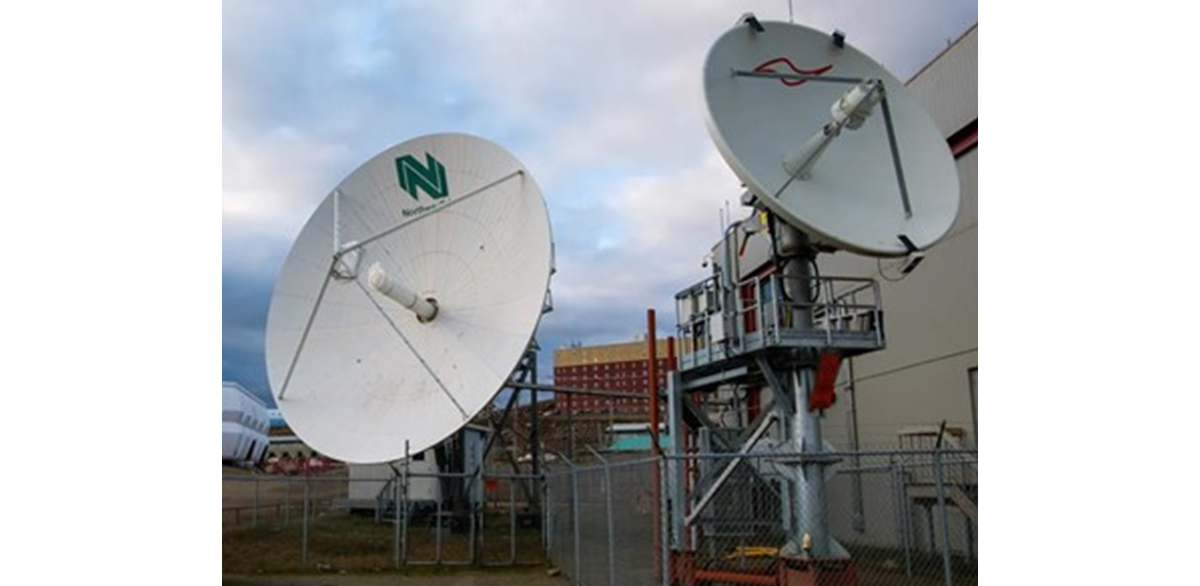
This high latency (>600 milliseconds) satellite dish in Iqaluit, Nunavut, is the leading telecommunication portal for the territorial capital: Nakasuk Elementary School and Inuksuk High School (left) and the Astro Hill complex (right) [30].

In Nunavut, the internet has low bandwidth, high latency, and poor reliability. It is expensive, making it difficult to do basic things like internet-based banking or real-time interactive social media encounters [30]. Figure 5 shows the high-latency satellite dish in Iqaluit, Nunavut.

A significant limitation in health care provision in northern Canada is the lack of a ubiquitous electronic health care record system. Many medical records in medical facilities across Canada are paper-based physical records supported by poorly connected laboratory and diagnostic imaging systems.

**Figure 6:**
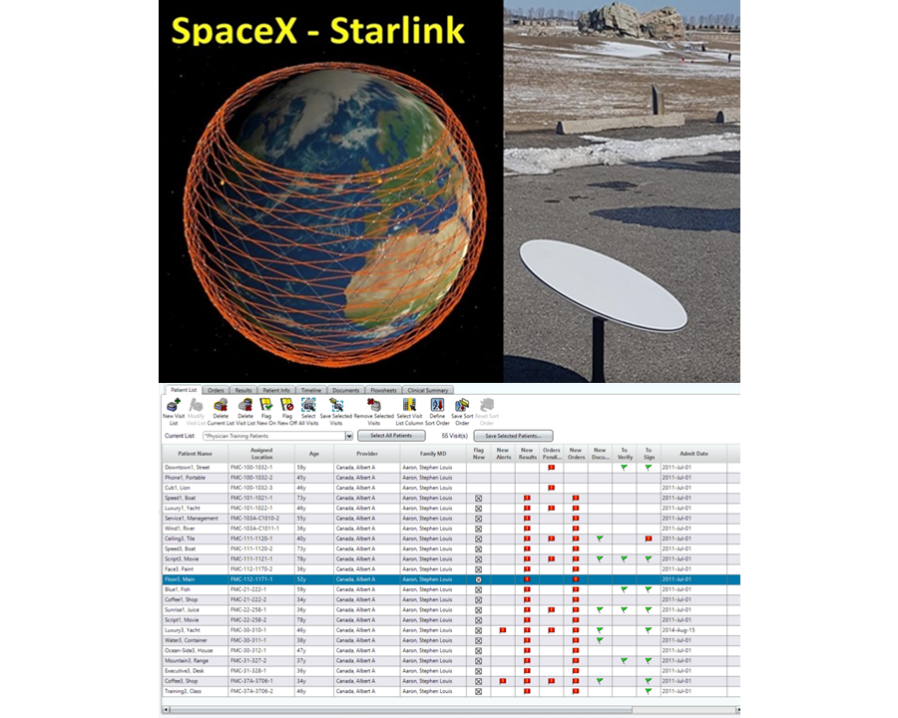
Using the SpaceX Starlink modem (upper panels), the telementored ultrasound (TMUS) team could log into the Allscripts Sunrise Clinical Manager training site by Citrix Workspace, examine patient records, and enter orders (lower panel).

In northern Canada, the population density is sparse, and the justification for deploying medical record systems in every community is currently not feasible. However, creating medical records for patients in northern Canada using a central electronic medical record system is now possible with the planned deployment of many commercial LEO-ComSats. The TMED team has recently demonstrated how simple and easy it is to access the electronic medical record systems currently deployed in Calgary, Alberta, using a SpaceX “Starlink” modem and Citrix Workspace (Figure 6). This demonstration confirms that a patient’s medical record could be accessed securely in real time anywhere in northern Canada.

**Figure 7:**
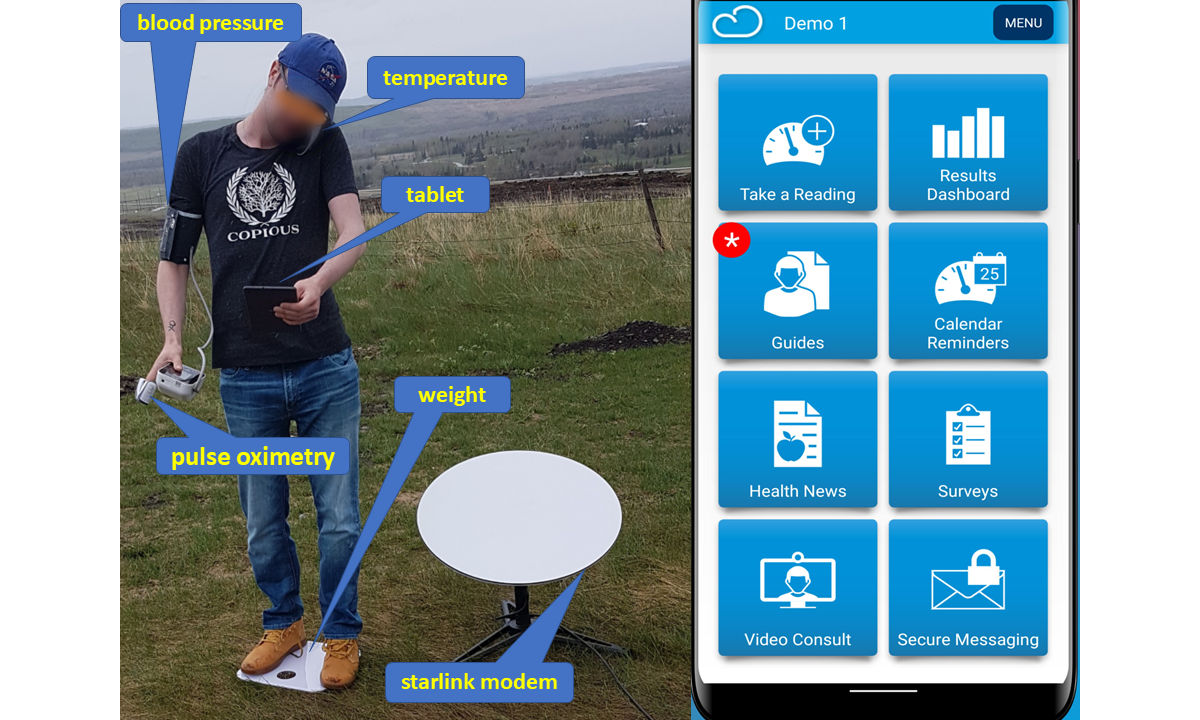
An untrained volunteer deployed the Cloud DX Connected Health Kit in a field with no mobile phone coverage and connected to the Cloud DX server using a SpaceX Starlink modem to provide blood pressure, heart rate, temperature, weight, and oxygen saturation to the Cloud DX Clinicians Portal.

The TMED team acquired a Cloud DX Connected Health Kit (Cloud DX) [31] and gave it to a volunteer without medical or information technology experience. The volunteer connected the Cloud DX components to a SpaceX Starlink modem powered by a Toyota RAV4 SUV in a field without cellular phone coverage. It took the volunteer 20 minutes to set up the devices. Within 10 minutes, the volunteer transmitted blood pressure, heart rate, temperature, weight, and oxygen saturation to a Cloud DX server in Toronto. The volunteer used the Connected Health tablet (available for Android and iOS devices), which includes an optional Telemedicine feature powered by Zoom to facilitate privacy and security-compliant video conference consultations from a Cloud DX application on the remote tablet to a Clinician’s Portal on the web (Figure 7)*.*

A remote web-based electronic medical record system could also recall diagnostic imaging in computed tomography or magnetic resonance imaging scans, ultrasound images, and x-rays acquired for local or remote medical experts to review, even though they are stored electronically elsewhere in Canada. Combining a ubiquitous electronic patient medical record through LEO-ComSat networks will significantly improve health care in northern Canada. This unified electronic medical record would document patient encounters with remote primary care physicians, nurses, and large tertiary care centers. For the first time, northern Canadian health care providers will have access to all their patient’s medical encounters from all medical facilities in Canada at any time.

Furthermore, a patient from northern Canada who receives treatment in a facility in the southern provinces can be confident that all the medical information documented during their encounter is immediately accessible by medical care providers in their northern community. This will be the first time continuity of care will be established over thousands of kilometers. Northern Canadian communities will now enjoy the same medical record resources that anyone living in a large Canadian city receives. Although these records will be managed over thousands of kilometers, they will still meet the rigid standards of security and privacy the Canadian government enforces by law, such as the Canadian Personal Information Protection and Electronic Documents Act (PIPEDA). Patients who live in northern Canada can be confident that their medical records will be treated with the same privacy and security as those who live in large Canadian cities.

Medical policy, standards, and requirements need to be created to allow a remote medical technician or nurse to telementor through a patient’s physical examination and accurately report the physical findings. Based on these standards, physician, nurse, and other health care provider training programs should include telementoring in their curriculums. Eventually, these policies, standards, and training programs to support telementored medical encounters should be sanctioned by the Colleges of Physicians and Surgeons in all provinces and the Canadian government. The Canadian health care system should rapidly establish policies and standards for our northern communities. These standards would allow medical and legal protective organizations (ie, the Canadian Medical Protective Association) to underwrite health care providers (individual, public, or corporate) in performing these online patient encounters to protect them from malpractice lawsuits. Without these legal protections for health care workers, providing online health care in northern Canada becomes extremely limited.

Over the past 2 years, the European Parliament and the European Council recently announced the EU4Health program to preserve online care use in Europe and Asia. This initiative promotes the sharing of digital health records, e-prescriptions, and telehealth [13]. Saudi Arabia is investigating the use of smartphone applications and a network to connect specialists with remotely located primary care centers and hospitals [13].

The ability to financially compensate remote and telementoring health care providers must also be addressed. Many capitation models reimburse health care providers for services, such as fee-for-service, episode-based payment, population-based payment, fixed salary, etc. Any of these methods would most likely be more affordable than the current paradigm of medical mass transport of patients over vast distances, isolating them from their family and community for at least a week and, in many circumstances, much more.

Finally, issues such as inter–province or territory medical-legal protection of health care providers and a government-sanctioned medical service fee structure for telementored medical encounters must be established rapidly. The telementoring concept should be immediately validated using LEO-ComSat links in latitudes of lower Canada. The resulting “proof of concept” will be directly applicable to promote the development of an improved and ubiquitous health care delivery model for remote and rural Canadians.

### Conclusion

**Figure 8:**
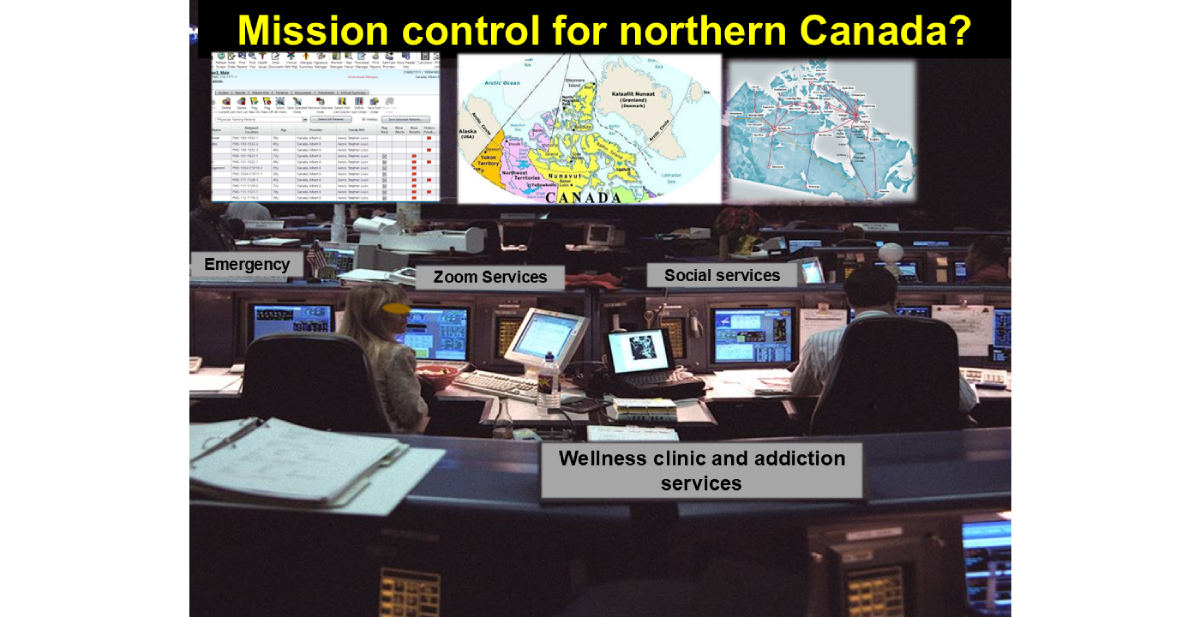
A hypothetical Canadian Medical Mission Control to “Connect the Unconnected” and “Support the Unsupported” by providing real-time or just-in-time medical support.

The deployment of LEO-ComSats could result in disruptive but positive changes in medical care for underserved communities in remote geographic locations across Canada. LEO-ComSats can be used to demonstrate the use of online medical encounters between a patient and a doctor in Canada, separated by thousands of kilometers. Most certainly, the academic medical centers in lower Canada could perform online telementored medical care to our northern communities, similar to the online care provided to many Canadians during the COVID-19 pandemic. Figure 8 shows a hypothetical Canadian Medical Mission Control.

This viewpoint was written to illustrate the need for effective design, testing, and validation of this new online and internet-based health care delivery model’s policies, standards, requirements, procedures, and protocols. Although the COVID-19 pandemic was the initial prime mover across all of Canada in the use of online medical encounters and creating rapidly devised reimbursement models, it was nonetheless created reactively, using real-time managerial fiat and poorly defined procedures based on minimal pedagogical experience, which made it “difficult to prove it was universally safe.” It is essential to proactively derive the medical policies, standards, and procedures for TMED and “prove it is safe” before LEO-ComSat technology is ubiquitously deployed in northern Canada.

## Abbreviations

AI: artificial intelligence
CSA: Canadian Space Agency
EHN: Edgeworth Health Network
ISS: International Space Station
LEO-ComSat: low Earth orbit communication satellite
NWT: Northwest Territories
PIPEDA: Personal Information Protection and Electronic Documents Act
TMED: telementored medicine
